# Evidence for external forcing of the Atlantic Multidecadal Oscillation since termination of the Little Ice Age

**DOI:** 10.1038/ncomms4323

**Published:** 2014-02-25

**Authors:** Mads Faurschou Knudsen, Bo Holm Jacobsen, Marit-Solveig Seidenkrantz, Jesper Olsen

**Affiliations:** 1 Centre for Past Climate Studies, Department of Geoscience, Aarhus University, Høegh-Guldsbergs Gade 2, Aarhus C DK-8000, Denmark; 2 Department of Physics and Astronomy, Aarhus University, Ny Munkegade 120, Aarhus C DK-8000, Denmark

## Abstract

The Atlantic Multidecadal Oscillation (AMO) represents a significant driver of Northern Hemisphere climate, but the forcing mechanisms pacing the AMO remain poorly understood. Here we use the available proxy records to investigate the influence of solar and volcanic forcing on the AMO over the last ~450 years. The evidence suggests that external forcing played a dominant role in pacing the AMO after termination of the Little Ice Age (LIA; *ca.* 1400–1800), with an instantaneous impact on mid-latitude sea-surface temperatures that spread across the North Atlantic over the ensuing ~5 years. In contrast, the role of external forcing was more ambiguous during the LIA. Our study further suggests that the Atlantic Meridional Overturning Circulation is important for linking external forcing with North Atlantic sea-surface temperatures, a conjecture that reconciles two opposing theories concerning the origin of the AMO.

## Abstract (web summary)

The Atlantic Multidecadal Oscillation strongly influences Northern Hemisphere climate, yet its primary driver is poorly understood. Knudsen *et al.* analyse proxy records from the past ~450 years and show that external forcing has dominated control of the oscillation since the termination of the Little Ice Age.

## Graphical abstract

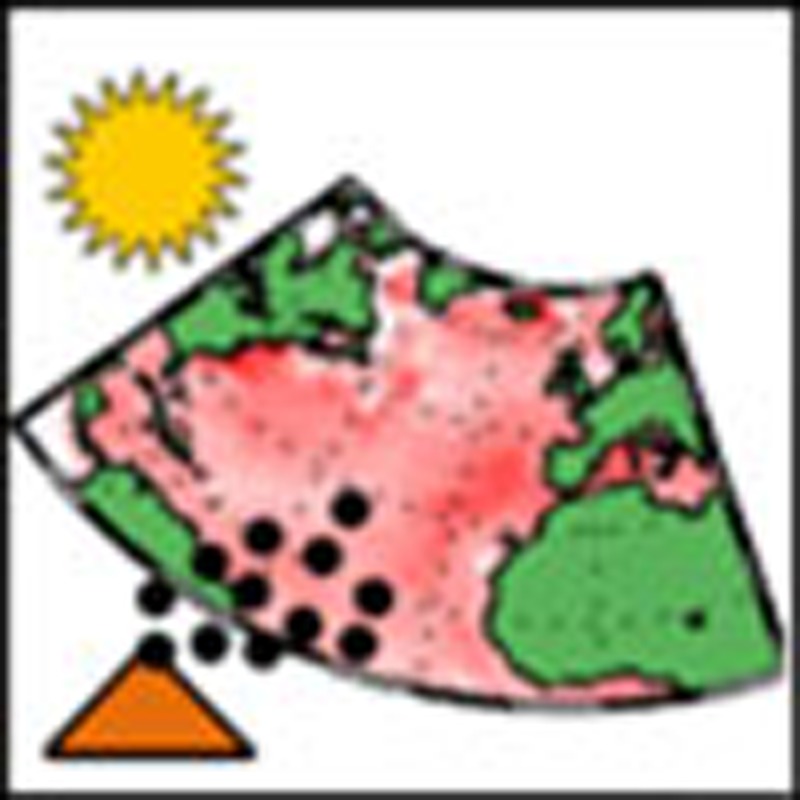

Variations in North Atlantic sea-surface temperatures (SSTs) are particularly prominent on multidecadal timescales. These changes, which exert a strong influence on climate in the North Atlantic region, are dominated by the alternation between warm and cold SST anomalies on a timescale of 60–80 years, a phenomenon known as the Atlantic Multidecadal Oscillation (AMO). The AMO has been identified in the instrumental record spanning the past ~150 years as a coherent, basin-wide pattern of oscillatory changes in SST^1^,^2^,^3^. The brevity of the instrumental record, however, renders it difficult to assess the nature of the AMO as well as the underlying forcing mechanisms. Climate proxy records from various geological archives show that the AMO variability extends several millennia back in time^4^,^5^,^6^,^7^,^8^, potentially providing an opportunity to improve our understanding of the possible forcing mechanisms driving the AMO.

The forcing mechanism pacing the AMO remains subject to considerable debate^9^. One school of thought holds that the AMO is driven by internal ocean variability and is related to multidecadal fluctuations in the Atlantic Meridional Overturning Circulation (AMOC). This notion is mainly based on climate model simulations with constant external forcing that exhibit multidecadal climate variability with a pattern and amplitude that resemble the observed AMO^10^,^11^. It is supported by studies of distinct AMOC fingerprints^12^,^13^, such as coherent, dipolar surface-subsurface temperature variations in the extratropical North Atlantic that are in-phase with the AMO. Additional supporting observations indicate that the dominant mode of variability throughout the last 8,000 years is incompatible with the known solar cycles^8^. In contrast, a recent modelling study concluded that the combined external forcing due to solar variability and volcanic eruptions has dictated the pace and phasing of the AMO over the past 600 years, as the combined solar and volcanic forcing is highly correlated to the AMO in the model with the forcing leading the AMO by ~5 years^9^. Other numerical experiments suggest that changes in total solar irradiance (TSI) alone may influence North Atlantic SSTs and hence the strength of the AMOC on multidecadal and centennial timescales^14^,^15^. Observational evidence for such a link is scarce, however, particularly on multidecadal timescales. Finally, it has also been argued that North Atlantic SSTs were driven by volcanic and anthropogenic emissions of aerosols throughout the past century^16^, particularly during the latter half of the twentieth century, but subsequent work casts considerable doubt on this claim because of considerable discrepancies between model results and observations^17^.

Identifying the relative roles of internal ocean variability and external forcing agents in driving multidecadal SST variability in the North Atlantic is important, in particular because the AMO purportedly influences climate variables of key importance to society, such as precipitation and hurricane activity^18^,^19^,^20^,^21^,^22^,^23^. In the present study we examine the relationship between the AMO and potential external forcing agents over the past 450 years based on statistical analyses of high-resolution proxy data. More specifically, we test whether the available proxy data support the hypothesis that North Atlantic SST variability was paced by solar and volcanic forcing over the past centuries, as claimed in a recent model-based study^9^. The evidence strongly supports the notion of external forcing of the AMO since the termination of the LIA, and analyses of the instrumental SST data indicate that the AMOC is important for linking changes in external forcing with North Atlantic SSTs. In contrast, the role of external forcing appears to have been more ambiguous during the LIA.

## Results

### Past AMO variability and changes in external forcing

**Figure 1:**
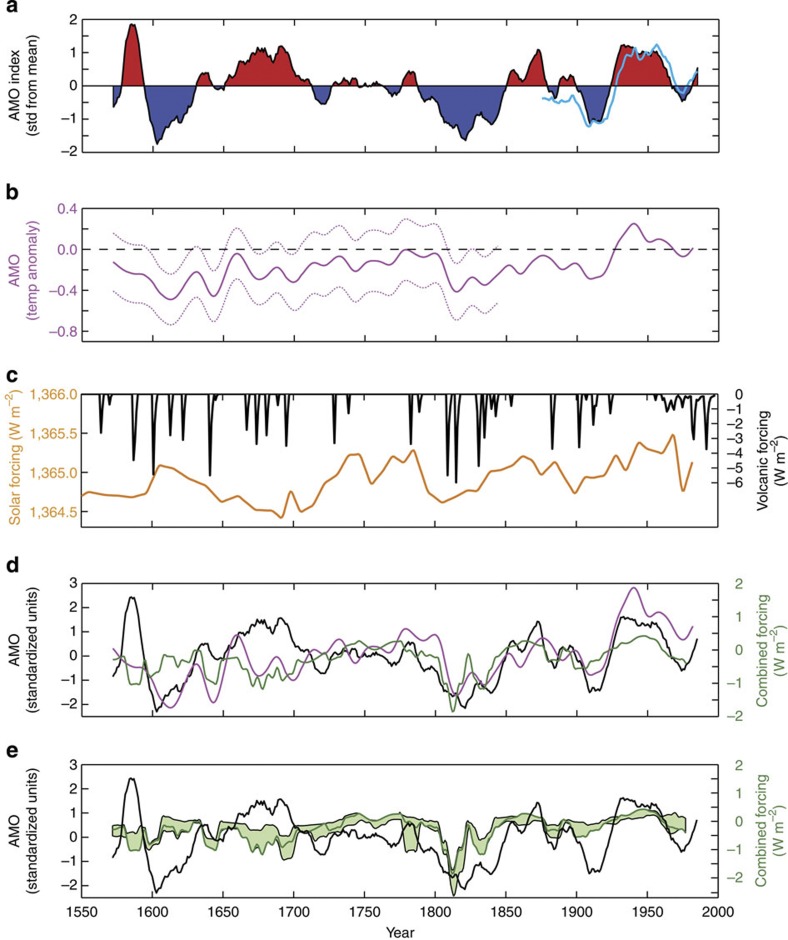
Past AMO variability and changes in solar and volcanic forcing. (**a**) Tree-ring-based AMO reconstruction^4^ (black line and red/blue fill) and the North Atlantic SST anomaly calculated from instrumental data^56^ (light blue line; scaled and subjected to an 11-year running mean). (**b**) A multiproxy-based AMO reconstruction and associated uncertainties^24^. (**c**) Changes in TSI^28^ (orange line) and volcanic forcing^30^ (black line). (**d**) Comparison between the tree-ring^4^ (black) and multiproxy^24^ (magenta) AMO reconstructions (standardized units) and changes in the combined solar and volcanic forcing based on the solar reconstruction of Delaygue and Bard^28^ and the volcanic reconstruction of Crowley^30^ (dark green line). (**e**) Comparison between the AMO reconstruction^4^ based on tree-rings (black line) and the external forcing envelope (light green shading) encompassing three different reconstructions of solar^28^,^31^,^32^ and volcanic^30^,^33^,^34^ forcing, respectively.

Various geological archives illustrate past variations in North Atlantic SSTs^24^, but only two genuine reconstructions of past AMO variability exist. A detailed reconstruction of the AMO requires that highly resolved proxy data from multiple sites in the North Atlantic region are combined into one record that can be reliably calibrated to the instrumental SST record. This, in turn, requires near-perfect age models in terms of accuracy and age uncertainties for all the included sites. One of the two AMO reconstructions that meet these criteria is based on 12 tree-ring records from eastern North America, Europe, Scandinavia and the Middle East^4^, which are regions with climates that currently covariate strongly with North Atlantic SST variability^18^,^19^,^25^ (Fig. 1a). This combined tree-ring record, which spans the period AD 1567–1990 (Fig. 1a), was calibrated to an instrumental record of North Atlantic SST anomalies^26^. The reconstruction subsequently exhibited significant skill in estimating SST anomalies outside the calibration period, attesting to the reliability of the reconstruction^4^. The annual resolution and near-optimal age control represent significant advantages of this AMO reconstruction, but the reliability of this reconstruction may be compromised if the centres of strong North Atlantic SST variability have shifted through time.

The other AMO reconstruction that can be argued to meet the criteria is based on a multiproxy approach that includes data from tree rings, ice cores, corals, speleothems and sediments^24^ (Fig. 1b). The strength of this reconstruction is the good geographical coverage and the robustness that a multiproxy approach provides. The downsides are the challenges associated with combining climate records from different archives and, most importantly, imperfect age control as several of the records suffer from large chronological uncertainties. The inclusion of data with relatively poor temporal resolution implies that only interdecadal and longer-term variations are meaningfully resolved^24^. Here we study these two existing reconstructions of past AMO variability during the time interval in which they overlap.

Various reconstructions of solar variability over the past 500 years exist. These generally resemble each other with respect to multidecadal trends. However, the amplitudes of grand solar maxima and minima, such as the Maunder Minimum (AD 1645–1715), are notoriously difficult to reconstruct and therefore give rise to some discrepancies among the available solar reconstructions^27^. In this study, we use a recent TSI reconstruction based on ice-core ^10^Be data from the South Pole and Dome Fuji^28^ (Fig. 1c), which was corrected for changes in the Earth’s magnetic field. These Antarctic ice cores are generally considered the most reliable recorders of past solar activity owing to the low degree of climatic noise influencing the deposition of meteoric ^10^Be (Baroni *et al.*^29^). With respect to past volcanic forcing, we apply the widely used record of Crowley^30^ (Fig. 1b). Similar to Otterå *et al.*^9^ in their reconstruction of long-term North Atlantic SST variability, we focus on the role of external forcing over the past centuries and therefore disregard changes in anthropogenic forcing, the effect of which was most pronounced over the past ~40 years.

### Relationship between the AMO and external forcings

**Figure 2:**
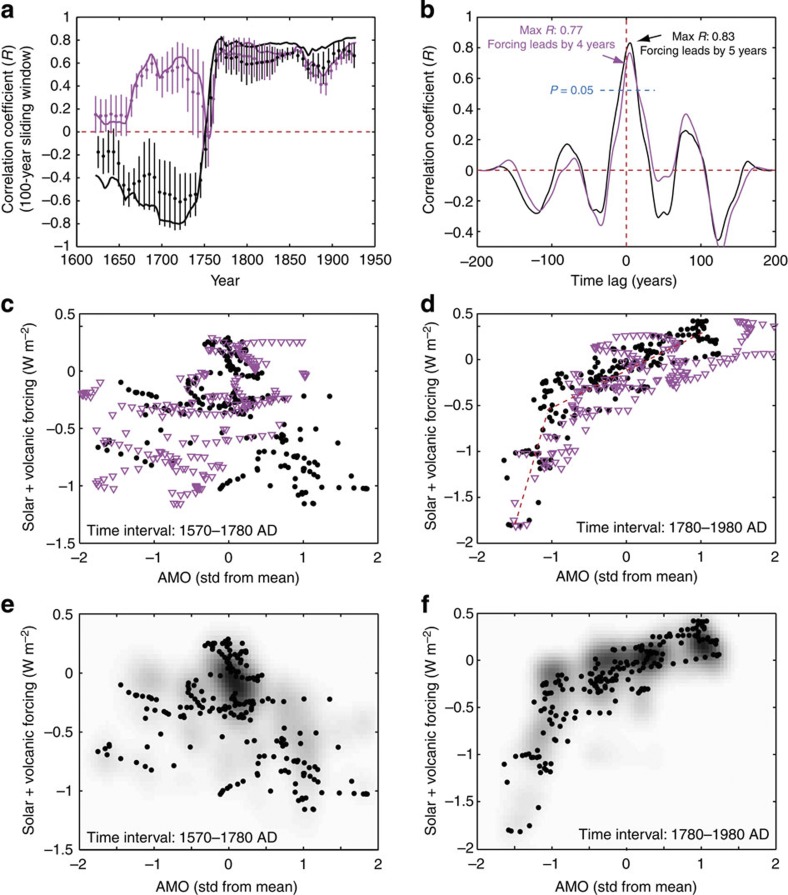
Relationships between external forcings and the AMO during the LIA and modern warming. (**a**) Changes in the Pearson correlation between the combined solar and volcanic forcing and the two AMO reconstructions for data in 100-year sliding windows. The solid lines show the running correlation obtained with the solar reconstruction of Delaygue and Bard^28^ and the volcanic record of Crowley^30^. The dots and thin vertical lines show the average running correlation obtained with the nine forcing combinations contained within the external forcing envelope and the associated 1σ s.d. (**b**) Cross-correlation between the AMO reconstruction and the combined solar and volcanic forcing for data after AD ~1775. Correlation coefficients above the dashed blue line are significant (*P*<0.05) when compared with 10,000 Monte Carlo simulations of red-noise AR1 data. Scatter plots of the reconstructed AMO and the combined external forcing before (**c**) and after (**d**) AD ~1775. All data included in **a**–**d** were smoothed using 11-year running means, and the black and magenta lines/dots/triangles denote the tree-ring^4^ and multiproxy^24^ AMO reconstructions, respectively. In **c** and **d**, the dashed red lines indicate the two-step linear trend between external forcing and the AMO. (**e**,**f**) Scatter plots of the tree-ring AMO reconstruction^4^ and the external forcing based on the solar reconstruction of Delaygue and Bard^28^ and the volcanic reconstruction of Crowley^30^ (black circles). The density plots (dark colours represent high densities) reflect the scatter obtained when comparing the tree-ring AMO with the envelope encompassing nine different combinations of solar and volcanic reconstructions (light green shading in Fig. 1e).

Our analyses show that the combined solar and volcanic forcing is highly correlated to both existing AMO reconstructions over the past two centuries. However, this relationship does not extend unchanged back in time (Fig. 1d). An abrupt change in correlation occurs around AD 1775, that is, roughly coinciding with the termination of the LIA (Fig. 2a). The correlation between the AMO reconstructions and the combined external forcing record is highly significant for both the tree-ring and multiproxy records (tree-ring AMO^4^: *R*=0.79 without lag correction and *P*~0.0003; multiproxy AMO^24^: *R*=0.70 without lag correction and *P*~0.006) after AD 1775 when compared with 10,000 randomly generated red-noise AR1 data. Remarkably, the AMO index^4^ based on tree rings appears anti-correlated to the combined forcing before AD ~1775 (Fig. 2a), although this anti-correlation is only marginally significant (*R*=−0.39, *P*~0.12). Comparison with red-noise AR1 time series suggests that the abrupt change in correlation around AD 1775 observed for the tree-ring AMO is highly unlikely to occur by chance (*P*~0.005). The multiproxy AMO reconstruction^24^ shows a distinct dip in the correlation around AD 1750 (Fig. 2a), where the AMO reconstruction and external forcing records are relatively flat. However, in contrast to the tree-ring-based AMO index, it still exhibits a positive, and marginally significant, correlation to the combined external forcing before AD ~1750 (*R*=0.46, *P*~0.07). Cross-correlation analyses furthermore show that both AMO reconstructions temporally lag the combined solar and volcanic forcing by ~5 years in the interval following the transition around AD 1775 (Fig. 2b), resulting in uniquely defined peaks in correlation at Δ*t*=5 years (*R*=0.83) and Δ*t*=4 years (*R*=0.77), respectively. In contrast, a more complex phasing is observed before the transition around AD 1775 where correlation peaks are found at variable time lags. This fundamental difference is also evident in scatter plots, suggesting a positive linear relationship between the AMO and external forcing after AD 1775 (Fig. 2d), and a poorly defined relationship before AD 1775 (Fig. 2c).

**Figure 3:**
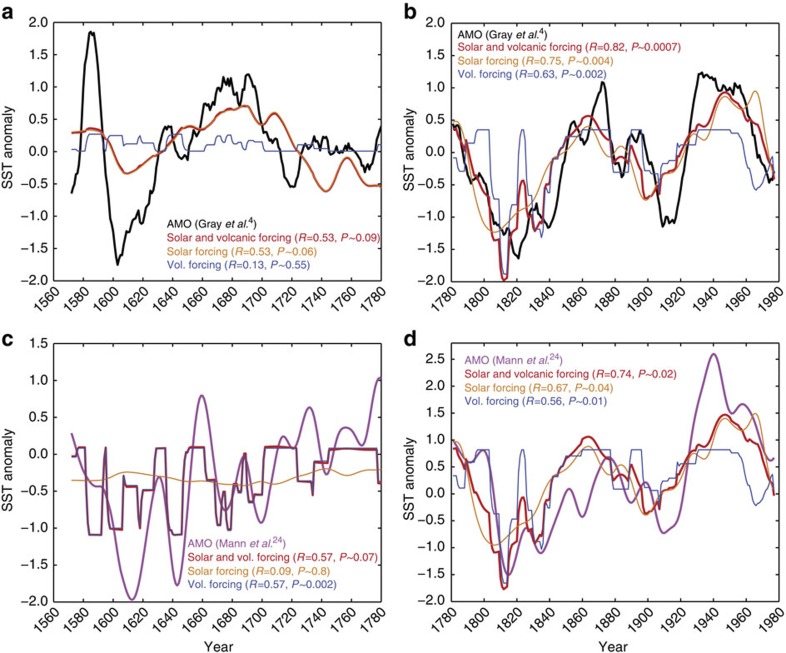
Linear regression analyses of the AMO and the external forcings. Multiple linear regression and linear regression analyses of the tree-ring AMO reconstruction^4^ and the external forcing records before (**a**) and after (**b**) AD ~1775. The blue line is the tree-ring-based AMO reconstruction, the red line is the best fit obtained through multiple linear regression analysis, while the orange and black lines represent the best fits obtained with solar forcing and volcanic forcing individually. The numbers indicate the probability of obtaining these optimal linear fits when compared with 10,000 Monte Carlo simulations of red-noise AR1 data. The regressions shown here were made with zero time lag (Δ*t*=0). Panels **c** and **d** are similar to **a** and **b** but for the multiproxy AMO reconstruction^24^ (magenta lines). Note that some lines lie on top of each other in **a** and **c**.

Multiple linear regression analyses show that the combined solar and volcanic forcing record provides a highly significant fit to both AMO reconstructions after AD 1775 (tree-ring AMO^4^: *R*=0.82 for Δ*t*=0; multiproxy AMO: *R*=0.74 for Δ*t*=0). For the tree-ring AMO^4^, the fit to the real AMO data is almost equal to the best fit obtained for any of the 10,000 red-noise AR1 time series used to estimate the significance of the regression model (*P*~0.0007; Fig. 3b), whereas the fit is slightly less significant (*P*~0.02) for the multiproxy AMO^24^ (Fig. 3d). Reasonable, but only marginally significant, fits between the combined forcing and the real AMO data are obtained for the interval before AD 1775 (*R*=0.53 and *P*~0.09; *R*=0.57; *P*~0.07; Fig. 3a,b). Linear regression analyses applying the TSI variability and volcanic forcing separately yield similar results for the two AMO reconstructions after AD 1775. For this period, both forcing records provide good, and reasonably significant, fits when applied individually to the AMO reconstructions (Fig. 3b,d), indicating that the solar and volcanic forcings were both positively aligned with changes in the AMO after *ca.* AD 1775. This is not true for the period before AD 1775 where the solar forcing provides a reasonably good fit to the tree-ring AMO reconstruction, while the linear regression analysis based solely on volcanic forcing reveals no relation at all to this AMO reconstruction (Fig. 3a). Note that the proportionality constant (*c*_TSI_) is negative before the transition around AD 1775 and positive after this transition, reflecting the change from negative correlation to positive correlation also observed in Fig. 2a. Interestingly, the volcanic forcing provides a good fit to the multiproxy AMO^24^ reconstruction before AD 1775, whereas the solar forcing shows no linear relationship at all with this multiproxy AMO reconstruction during this period (Fig. 3c).

The overall relationship between the AMO and the combined external forcing is largely independent of the exact choice of forcings used in the analyses. The forcing envelope used in this study (light green band in Fig. 1e), encompassing nine different combinations of solar^28^,^31^,^32^ and volcanic^30^,^33^,^34^ reconstructions, display similar relationships to the AMO both before (Fig. 2e) and after (Fig. 2f) the transition around AD 1775. Solar reconstructions characterized by relatively small amplitudes during grand minima and maxima give rise to a slightly smaller slopes compared with that obtained using the solar reconstruction of Delaygue and Bard^28^ (density plot compared with black circles in Fig. 2f). Moreover, the volcanic forcing associated with the Laki eruptions around AD 1783 is particularly strong in the volcanic reconstruction of Gao *et al.*^33^ compared with other reconstructions, and hence in three of the nine combined forcing records used in this study. This forcing anomaly around AD 1783, which coincides with a warm AMO phase, explains the moderate grey shading outside the well-defined two-step linear trend (Fig. 2f).

### External forcing of the AMO since the LIA

The close relationship between both AMO reconstructions and the combined solar and volcanic forcing after ca. AD 1775 (Figs 2 and 3) lends strong observational support to the climate model simulations by Otterå *et al.*^9^ In these model simulations, the AMO lags the external forcing by ~5 years, which is in close agreement with the ~5-year lag observed in the present study for the period after AD 1775. A more detailed explanation for this lagged North Atlantic SST response to solar variability was recently proposed based on idealized experiments showing that a step change in ultraviolet forcing has an immediate impact on the atmosphere, which subsequently takes several years to accumulate in the ocean^35^. During this time, the atmospheric response continues to increase, suggesting a positive feedback between the ocean and atmosphere. Similarly, several studies indicate that the ‘top-down’ stratosphere–troposphere mechanism represents an important response to solar variability, particularly at high latitudes. However, the magnitude of this effect is uncertain, in part, because past changes in the ultraviolet region of the solar irradiance spectrum are poorly constrained as satellite observations of spectral irradiance cover less than one solar cycle^36^. The combined solar and volcanic forcing applied in the present study (in W m^−2^) therefore provides a simplistic representation of the external forcing that, similar to most other studies, underestimates indirect mechanisms between external forcing and climate. Nevertheless, we note that the regression models for both solar and volcanic forcings provide significant fits to both AMO reconstructions after AD ~1775 (Fig. 3b), suggesting that these forcings are highly consistent with AMO variability during this period. This strongly supports a link between the AMO and solar as well as volcanic forcing for the period after AD ~1775 that scales linearly with the reconstructed changes in W m^−2^.

**Figure 4:**
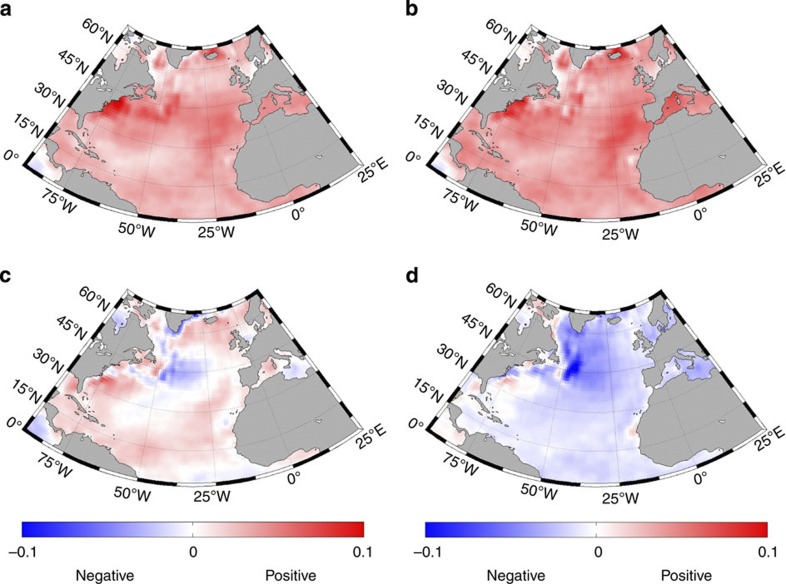
Spatial relationship between external forcings and instrumental North Atlantic SSTs. Cross-covariances between instrumental North Atlantic SSTs obtained from HadISST^56^ and the combined solar and volcanic forcing (Fig. 1d) between 1870 and 1982 for time lags of 0 years (**a**), 5 years (**b**), 20 years (**c**) and 30 years (**d**). The colour bar showing the covariance (in °C × W m^−2^) is the same for all panels.

Covariance analyses between spatially resolved instrumental SST data from the North Atlantic and the combined solar and volcanic forcing (Fig. 4) may provide insight into how North Atlantic SSTs responded to changes in the forcing, in particular the role of the AMOC that is known to have a strong impact on North Atlantic SSTs^37^,^38^,^39^. For zero time lag, positive covariances are mainly observed off the US east coast and across the North Atlantic at mid-latitudes, but also west of Spain and North Africa, and across the subtropics (Fig. 4a), corresponding to the main path of the Gulf Stream and the North Atlantic Subtropical Gyre region. Similarly, positive covariances are observed almost throughout the North Atlantic for a time lag of 5 years (Fig. 4b), which is in good agreement with the cross-correlation between the combined forcing and the reconstructed AMO (Fig. 2b). This suggests that parts of the North Atlantic responds almost instantaneously to the forcing, possibly via a link between the North Atlantic Oscillation (NAO) and Gulf Stream SSTs off the east coast of the United States, and it takes about 5 years for this change to spread and swell to its maximum throughout the North Atlantic, the Mediterranean and the Caribbean. A zone of negative covariances emerges in the central leg of the Gulf Stream and in the Subpolar Gyre region south of Greenland and west of Newfoundland for a time lag of 20 years (Fig. 4c), suggesting that this might be where the SST instability starts in the transition from a warm phase to a cold phase. Finally, negative covariances are observed throughout the North Atlantic for a lag of 30 years (Fig. 4d), which is expected as such a time lag corresponds to half the periodicity of the AMO. The negative covariances reach their maximum west of Newfoundland where the transition initiated. In conclusion, both the strongest near-instant response of the SST (Δ*t*=0) to external forcing and the area with the strongest negative anomalies for time lags of 20 and 30 years are found at the location of the core of the Gulf Stream, whereas the effect of the forcing gradually spreads over the North Atlantic region following the paths of the subpolar and subtropical gyres. This strongly suggests that the AMOC plays a major role in linking external forcing with North Atlantic SSTs.

## Discussion

While the relationship between the AMO reconstructions and the combined external forcing was unambiguous after AD ~1775, the relationship appears to have been more complicated before this transition. The period before AD 1775 corresponds largely to the later part of the LIA, a cold period of the Northern Hemisphere during which both the atmospheric and ocean circulation, including the AMOC, were different compared with conditions during the ensuing modern warming and today^40^,^41^.

A number of studies have suggested a generally weaker AMOC during the LIA compared with today^41^,^42^,^43^, resulting in a generally weaker northward heat transport and decreasing North Atlantic SSTs at high northern latitudes^43^,^44^,^45^. The cause of this weakening of the AMOC during the LIA has been much debated, but it appears to be linked to a predominantly negative NAO^40^,^41^,^46^,^47^,^48^,^49^ phase causing a weakening of the Northern Hemisphere westerlies. This may have been combined with an El Niño-like state in the Pacific region causing a northward shift in the Southern Hemisphere westerlies, thereby decreasing the exchange of water between the Indian Ocean and the South Atlantic^50^,^51^,^52^. The cooling of the Northern Hemisphere also resulted in a southward migration of the Intertropical Convergence Zone^53^,^54^.

Before the transition around the termination of the LIA, the two AMO reconstructions indicate almost opposite relations to external forcing. The most notable discrepancy between the two AMO reconstructions coincides in time with the Maunder Minimum, a period during which the tree-ring AMO^4^ indicates a positive AMO phase, which results in the marginally significant negative relationship with solar forcing (Fig. 3a). Contrary to this, the multiproxy AMO^24^ shows no relationship with solar forcing during the LIA, but a potentially close relationship with volcanic forcing (Fig. 3c). The regression analysis indicates similar relationships between volcanic forcing and the multiproxy AMO before and after the transition around AD 1775. Remarkably, this appears to be the most consistent relationship between one of the external forcing components and the AMO reconstructions throughout the last 450 years, which underlines the potential importance of volcanic forcing as a driver of North Atlantic SST variability.

The large-scale climatic and oceanographic changes that took place around the termination of the LIA strongly affected North Atlantic SSTs, although these changes were not coherent across the entire North Atlantic (Sicre *et al.*, manuscript in preparation). These geographical differences in the response of North Atlantic SST may in part explain the difference between the two AMO reconstructions. The limited geographical coverage of the tree-ring AMO reconstruction^4^ makes this reconstruction particularly sensitive to such changes, and potentially a less reliable recorder of past AMO variability, in comparison with the multiproxy AMO reconstruction. It is also possible that other aspects of the calibration of the proxy records to the instrumental record were invalid during the LIA as a consequence of a varying relationship between SSTs and the proxy archives owing to overall differences in climate, especially the shift in NAO mode. The tree-ring AMO is more prone to such calibration problems as it relies on a narrowly defined relationship between SSTs and tree rings. The multiproxy AMO is presumably less prone to such changes affecting the calibration over time, but the simultaneous calibration of records from different archives generally implies large uncertainties (Fig. 1b). The role of external forcing during the LIA, in particular the transition around AD 1750–1775, therefore remains uncertain.

Despite the differences between the two AMO reconstructions, it is evident that the unambiguous link observed between AMO variability and the combined solar and volcanic forcing after AD 1775 is not present before AD 1750. As outlined above, we conjecture that the imprint of the external forcing on the AMO is propagated throughout the North Atlantic region via the AMOC (Fig. 4). If this proves correct, the weaker AMOC that characterized the LIA^41^,^42^,^43^ would imply a weaker response of ocean circulation, and hence imprint on North Atlantic SSTs, to external forcing. It is thus possible that the strength of the AMOC may have modulated the significance of the imprint of variations in external forcing on the AMO throughout the last 8,000 years during which the AMO signal can be traced^8^.

## Methods

### Data and correlation analyses

To investigate the relationship between the AMO, solar and volcanic forcing, we combined the two forcing records by adding them year by year (in W m^−2^). The irregularly sampled solar reconstruction based on ^10^Be in Antarctic ice cores was interpolated to obtain an annual time resolution similar to the timescale of other records. An 11-year running mean was subsequently calculated for the AMO reconstructions and the combined solar and volcanic forcing. Sensitivity tests ensured that application of such a symmetric filter to all data sets does not introduce any artificial leads/lags during cross-correlation analyses. Pearson’s correlation and cross-correlation coefficients were then calculated for the entire length of the AMO reconstruction and for data in 100-year sliding windows (Fig. 2a), that is, nine degrees of freedom per window.

### Evaluation of statistical significance

Synthetic red-noise data were generated to evaluate the statistical significance of the correlations obtained between the combined forcing and the two AMO reconstructions. These data were modelled as an AR1 process following the procedure of Schulz and Mudelsee^55^ with characteristic memory factors (ϕ) equal to those obtained for the annual AMO data associated with the tree-ring-based reconstruction and the multiproxy-based reconstruction, respectively. The characteristic memory factors (ϕ) were obtained using the covariance function for the AR1 process given as cov( X(*t*_i_), X(*t*_j_) )=Φ_0_ × ϕ ^|j−i|^, where Φ_0_ is the variance and cov( X(*t*_i_), X(*t*_j_)) is the covariance observed at |j−i| time steps from zero lag. Following their computation, these annual synthetic data were subsequently subjected to an 11-year running mean. A total of 10,000 synthetic AMO data sets were simulated for both AMO reconstructions using a Monte Carlo approach and the correlation coefficients obtained between the synthetic AMO data and the combined forcing were compared with those obtained for the real AMO reconstructions. These correlations, which were computed for the LIA (before AD 1775) and modern warming (after AD 1775) separately, were used to estimate the probability (*P*) of obtaining the high correlations between external forcing and the AMO by chance. Similarly, 10,000 red-noise time series were additionally computed for the whole time period to estimate the statistical significance of the distinct shift in correlation observed for the tree-ring AMO (Fig. 2a). This was done simply by observing how often a shift from −0.8 to 0.8 (or similar magnitude) in the Pearson correlation coefficient occurred within 100 years (a conservative estimate) in the 100-year running correlation analyses of the simulated time series.

### Multiple linear regression analyses

Multiple linear regression and linear regression models were used to estimate the degree to which changes in solar and volcanic forcings may fit the reconstructed AMO record during various time intervals within a linear scaling framework. The North Atlantic SST anomaly (SSTA_R_) is reconstructed as a function of time, SSTA_R_(*t*)*=c*_0_*+c*_TSI_ TSI(*t–*Δ*t*_TSI_)*+c*_V_ *V*(*t*–Δ*t*_V_), where TSI is the total solar irradiance, *V* is the volcanic forcing and *c* the various proportionality constants. Analyses were made using both the combined forcing, the solar and volcanic forcings individually, and with zero lag as well as time lags based on the cross-correlation between the AMO reconstructions and changes in the forcings. The fitted coefficients were obtained through multiple linear regression against the reconstructed AMO data using least-squares inversion (***m***_est_*=*(***G***^T^ ***G***) ***G***^T^ ***d***_obs_). This procedure was additionally applied to the 10,000 red-noise AR1 data sets to investigate the significance of the fit obtained using linear regression models. The resulting probabilities (*P*) are used to assess the potential roles of solar and volcanic forcing, both combined and individually, in driving AMO variability over time.

### External forcing envelope

To assess how sensitive the relationship between the AMO and the external forcing is to the choice of solar and volcanic reconstructions, we constructed an external forcing envelope based on three different reconstructions of solar variability, based on ^10^Be (Delaygue and Bard^28^), ^14^C (Bard *et al.*^31^) and sunspots^30^,^32^ respectively, and three different reconstructions of changes in volcanic forcing^30^,^33^,^34^, respectively. The envelope encompassing the resulting nine possible external forcing records is shown in Fig. 1e (light green shading). A running correlation based on 100-year time windows was calculated for all forcing combinations included in the envelope, and the average correlation and associated s.d. were computed (Fig. 2a). The external forcing records were also plotted against the tree-ring AMO reconstruction^4^. The results are shown as density plots in Fig. 2e,f, indicating that the relationship between the AMO and external forcing is largely insensitive to the choice of forcing reconstructions. Comparisons of the Pearson correlation and multiple linear regression analyses with similar analyses of red-noise time series support the overall conclusions.

## Author contributions

M.F.K. designed the study. The methodological approach and computations were developed and carried out by M.F.K. and B.H.J. All authors contributed to the interpretation of the results. M.F.K. wrote the manuscript with significant input from B.H.J., M.-S.S. and J.O.

## Additional information

**How to cite this article:** Knudsen, M. F. *et al.* Evidence for external forcing of the Atlantic Multidecadal Oscillation since termination of the Little Ice Age. *Nat. Commun.* 5:3323 doi: 10.1038/ncomms4323 (2014).
